# Supplementary material for: Molecular bases for HOIPINs-mediated inhibition of LUBAC and innate immune responses
Source: Commun Biol. 2020 Apr 3;3:163. doi: 10.1038/s42003-020-0882-8 (PMC7125101; doi:10.1038/s42003-020-0882-8)
Supplement: Supplementary file 1 — Supplemental Information [file 42003_2020_882_MOESM1_ESM.pdf]

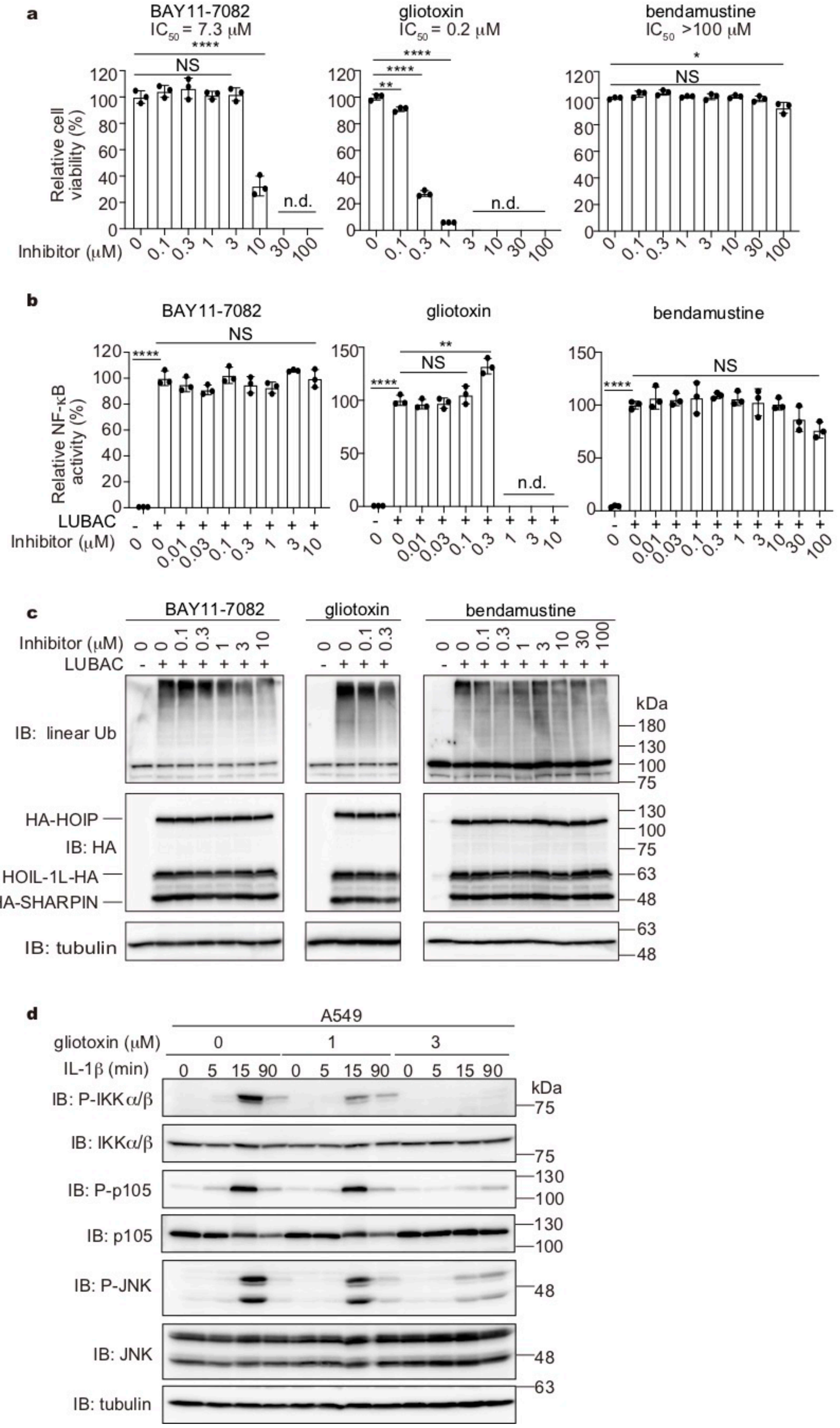

Supplementary Fig. 1 Effects of reported LUBAC inhibitors on cytotoxicity, NF-κB activation, and linear ubiquitination. **a** BAY11-7082 and gliotoxin are cytotoxic. A549 cells were treated with the indicated concentrations of BAY11-7082,

gliotoxin, or bendamustine for 72 h, and the cell viability was assessed by a CellTiter-Glo luminescent cell viability assay. Taking the cell viability without inhibitors as 100%, the relative cell viabilities are indicated. **b, c** Previously reported LUBAC inhibitors do not suppress LUBAC-induced NF-κB activation and linear ubiquitination. HA-tagged LUBAC subunits and NF-κB luciferase reporter were co-transfected into HEK293T cells, which were treated with the indicated concentrations of compounds for 24 h. The relative NF-κB luciferase activities (**b**) and the immunoblotting of intracellular linear ubiquitin (**c**) are shown. **d** Gliotoxin suppresses the NF-κB and MAPK pathways. A549 cells were pre-treated with the indicated concentrations of gliotoxin for 30 min, and stimulated with 1 ng/ml IL-1β with sustained treatment with gliotoxin for the indicated period. The cells were then lysed and immunoblotting was performed with the indicated antibodies. In (**a**, **b**), data are shown as means ±SEM, n=3, NS: not significant, n.d.: not determined, *P<0.05, **P <0.01, ****P <0.0001, one-way ANOVA with Tukey's post-hoc test.

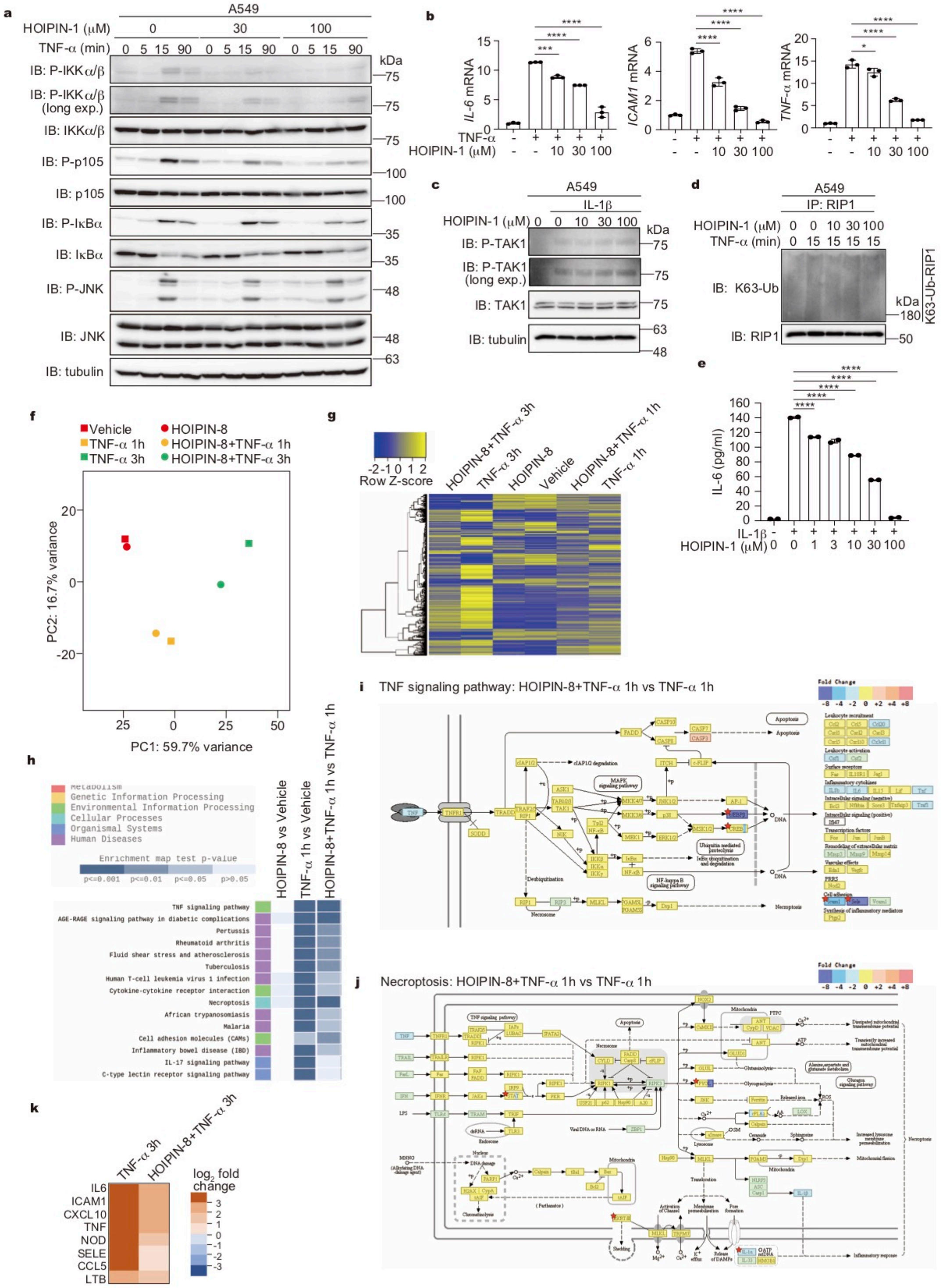

Supplementary Fig. 2 HOIPINs inhibit inflammatory cytokine-induced canonical NF-κB activation. **a** HOIPIN-1 inhibits TNF-α-induced NF-κB activation. A549 cells were pre-treated with the indicated concentrations of HOIPIN-1 for 30 min,

and stimulated with 10 ng/ml TNF-α with sustained treatment with HOIPIN-1. Cell lysates were immunoblotted with the depicted antibodies. **b** Reduced expression of NF-κB targets by HOIPIN-1. A549 cells were pre-treated with the indicated concentrations of HOIPIN-1 for 3 h, and treated with 10 ng/ml TNF-α for 2 h with HOIPIN-1. The mRNA levels of *IL-6*, *ICAM1*, and *TNF-α* were assessed by qPCR. Data are shown as means ±SEM, n=3. **c** HOIPIN-1 shows no effect on TAK1 activation. A549 cells were stimulated with 1 ng/ml IL-1β in the presence of the indicated concentrations of HOIPIN-1, and cell lysates were immunoblotted by the indicated antibodies. **d** K63-linked ubiquitination of RIP was not affected by HOIPIN-1. A549 cells were pre-treated with the indicated concentrations of HOIPIN-1 for 30 min, and stimulated with 1 μg/ml FLAG-TNF-α and HOIPIN-1 for 10 min. After immunoprecipitation with an anti-RIP1 antibody, immunoblotting analyses were performed using the indicated antibodies. **e** Reduced secretion of IL-6 by HOIPIN-1. A549 cells were treated with the indicated concentrations of HOIPIN-1 and 1.5 ng/ml IL-1β for 24 h, and the secreted IL-6 from two biological replicates was quantitated by ELISA. **f-k** The TNF-α-induced gene expression in A549 cells was suppressed by HOIPIN-8. A549 cells were stimulated with 10 ng/ml TNF-α, in either the absence or presence of 30 μM HOIPIN-8 for 1 h or 3h. The cells were lysed, and subjected to transcriptome-wide expression using their extracted total RNA as in Fig. 1g-i. A principal component analysis of the RNA-seq analysis (**f**), the heatmaps of the gene expression in the inflammatory pathway (**g**), top 15 terms in KEGG enrichment analysis (**h**), KEGG pathway maps on TNF signaling (**i**) and necroptosis (**j**), and the major genes in the NF-κB signal (**k**) are shown. In (**b**, **e**), *P<0.05, ***P <0.001, ****P <0.0001, one-way ANOVA with Tukey's post-hoc test.

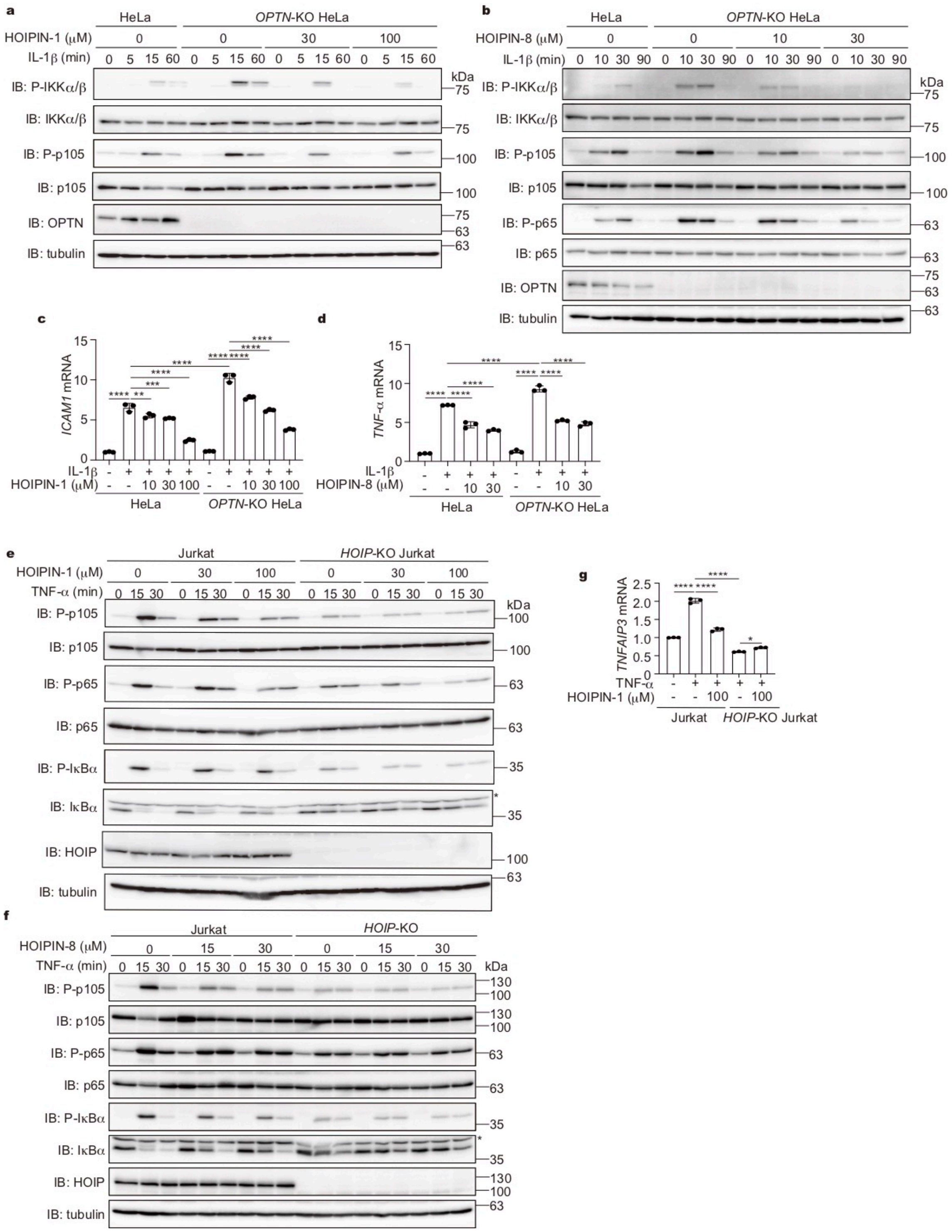

Supplementary Fig. 3 Effects of HOIPINs on *OPTN*- and *HOIP*-deficient cells. **a, b** The elevated NF-κB activity in *OPTN*-deficient HeLa cells is suppressed by HOIPINs. Parental and *OPTN*-deficient HeLa cells were pre-treated with the indicated concentrations of HOIPIN-1 (**a**) or HOIPIN-8 (**b**) for 30 min. The cells

were then stimulated with 1 ng/ml IL-1β in the presence of HOIPINs for the indicated period. Cell lysates were immunoblotted with the depicted antibodies. **c**, **d** Reduced expression of NF-κB-target genes in *OPTN*-deficient HeLa cells by HOIPINs. Parental HeLa cells or *OPTN*-deficient HeLa cells were pre-treated with the indicated concentrations of HOIPIN-1 or -8 for 3 h, and stimulated with 1 ng/ml IL-1β with HOIPINs for 1 h. Afterwards, qPCR analyses of *ICAM1* (**c**) and TNF-α (**d**) were performed. **e, f** The inhibitory effects of HOIPINs on NF-κB activation are canceled in *HOIP*-deficient Jurkat cells. WT- and *HOIP*-deficient Jurkat cells were pre-treated with the indicated concentrations of HOIPIN-1 (**e**) or HOIPIN-8 (**f**) for 30 min, and stimulated with 50 ng/ml TNF-α for the indicated period. The cell lysates were then immunoblotted with the depicted antibodies. **g** qPCR analysis for TNF-α- and HOIPIN-1-treated parental Jurkat or *HOIP*-deficient Jurkat cells. Cells were pre-treated with the indicated concentrations of HOIPIN-1 for 2 h, and stimulated with 50 ng/ml TNF-α for 1 h, and then the qPCR analysis was performed. In (**c**, **d**, **g**), data are shown as means ±SEM, n=3, NS: not significant, *P<0.05, **P <0.01, ***P <0.001, ****P <0.0001, one-way ANOVA with Tukey's post-hoc test.

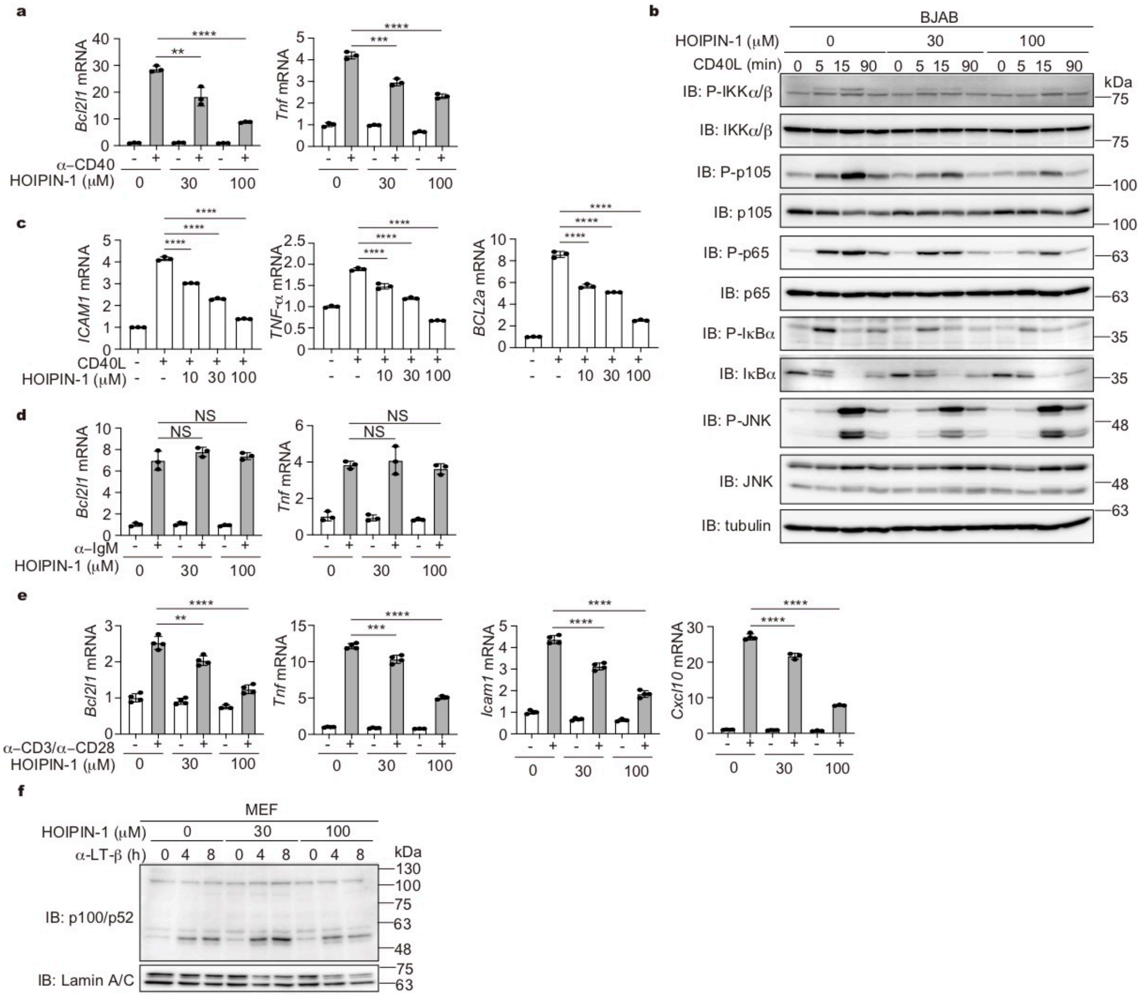

Supplementary Fig. 4 HOIPIN-1 attenuates LUBAC-associated NF-κB pathways. **a** HOIPIN-1 suppresses the CD40-mediated NF-κB activation pathway. Mouse splenic B cells were pre-treated with the indicated concentrations of HOIPIN-1 for 30 min, and then stimulated with 3 μg/ml anti-CD40 antibody in the presence of HOIPIN-1 for 3 h (*Bcl2l1*) or 1h (*Tnf-α*). The expression levels of the mRNAs were analyzed by qPCR. **b** Suppression of the CD40-mediated NF-κB activation pathway by HOIPIN-1. BJAB cells were pre-treated with the indicated concentrations of HOIPIN-1 for 30 min, and then stimulated with 1 μg/ml CD40L in the presence of various concentrations of HOIPIN-1 for the indicated period. The immunoblotting was performed with the indicated antibodies. **c** Reduced expression of NF-κB targets from CD40L-stimulated BJAB cells. BJAB cells were pre-treated with the indicated concentrations of HOIPIN-1 for 2 h, and then stimulated with 1 μg/ml CD40L for 3 h with HOIPIN-1. The expression of NF-κB

target genes was examined by a qPCR analysis. **d** HOIPIN-1 does not suppress the BCR-mediated pathway. Mouse splenic B cells were analyzed as in (**a**), but the cells were stimulated with 5 μg/ml anti-IgM antibody. **e** Reduction of TCR-mediated expression of NF-κB targets by HOIPIN-1. Mouse splenic T cells were pre-treated with the indicated concentrations of HOIPIN-1 for 30 min, and then stimulated with 5 μg/ml anti-CD3 and 1 μg/ml anti-CD28 antibodies for 3 h. The mRNA levels of NF-κB-targets were assessed by qPCR. **f** HOIPIN-1 does not inhibit the lymphotoxin (LT)-β-induced non-canonical NF-κB pathway. MEFs were pre-treated with the indicated concentrations of HOIPIN-1 for 30 min, and stimulated with anti-LTβR mAb (0.3 μg/ml) for the indicated period. Cells were lysed with NE-PER reagents (Thermo), and the nuclear fraction was immunoblotted with the indicated antibodies. In (**a**, **c**, **d**, **e**), data are shown as means ± SEM, n=3, NS: not significant, $^{**}P$ <0.01, $^{***}P$ <0.001, $^{****}P$ <0.0001, one-way ANOVA with Tukey's post-hoc test.

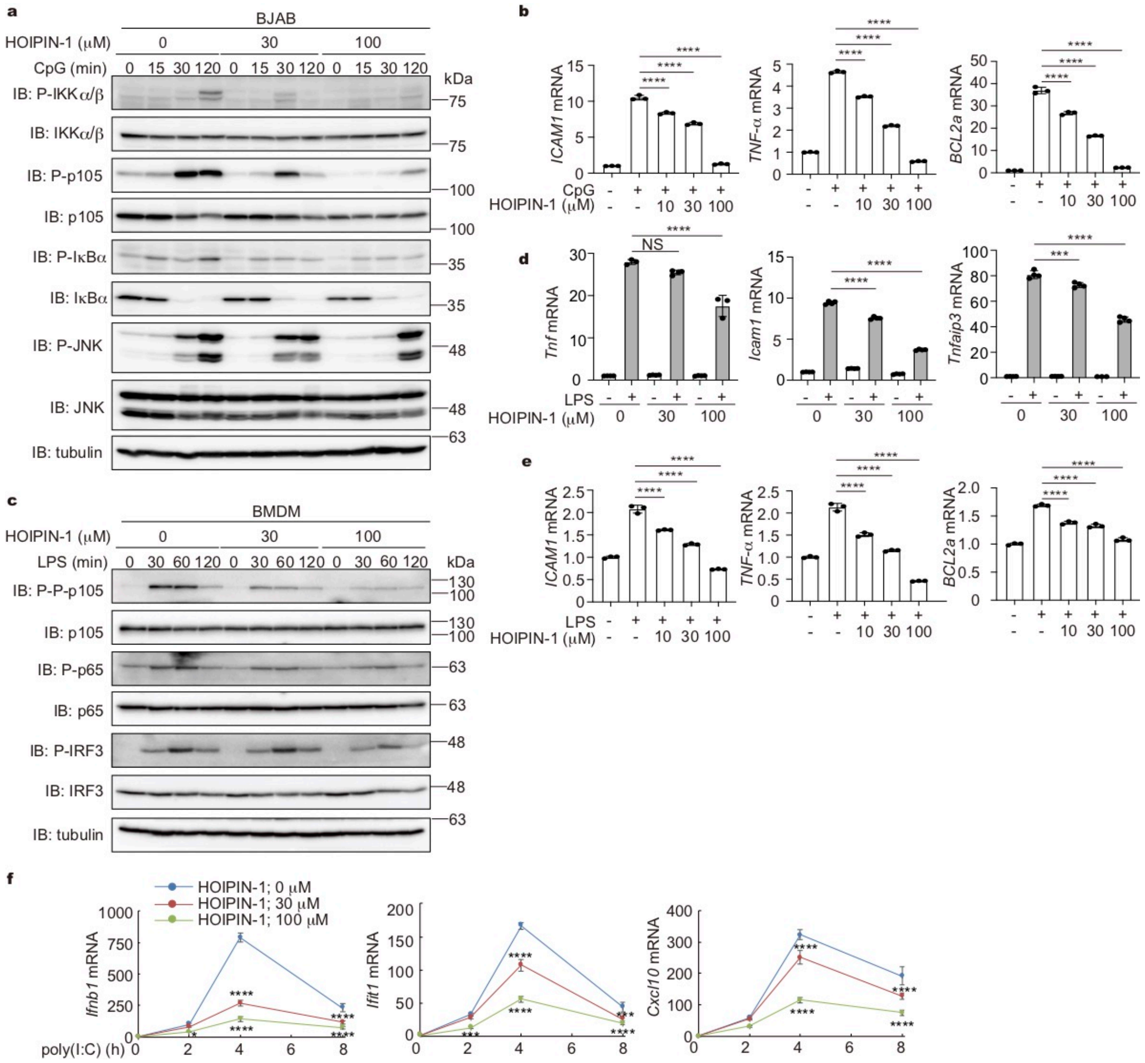

Supplementary Fig. 5 Various PAMPs-induced NF-κB and IFN antiviral pathways are inhibited by HOIPIN-1. **a** CpG-induced NF-κB activation is suppressed by HOIPIN-1. BJAB cells were pre-treated with the indicated concentrations of HOIPIN-1 for 30 min, and stimulated with 3 μM CpG in the presence of HOIPIN-1. The cell lysates were then immunoblotted with the depicted antibodies. **b** HOIPIN-1 suppresses CpG-mediated NF-κB activation. BJAB cells were pre-treated with the indicated concentrations of HOIPIN-1 for 2 h, and then stimulated with 1 μM CpG for 3 h. The mRNA levels of NF-κB targets were assessed by qPCR. **c** Suppressed phosphorylation of NF-κB and antiviral signaling factors. BMDM cells were pre-treated with the indicated concentrations of HOIPIN-1 for 30 min, and stimulated with 20 μg/ml LPS, and then the cell lysates were immunoblotted with the indicated antibodies. **d** The LPS-induced

NF-κB pathway is suppressed by HOIPIN-1. Mouse BMDM cells were pre-treated with the indicated concentrations of HOIPIN-1 for 30 min, and then stimulated with 100 ng/ml LPS for 1 h with HOIPIN-1, and the mRNA levels induced by NF-κB were assessed by qPCR. **e** HOIPIN-1 suppresses LPS-mediated NF-κB activation. BJAB cells were treated and the qPCR analysis was performed as in (**b**), except that the cells were stimulated with 1 μg/ml LPS. **f** HOIPIN-1 inhibits the poly(I:C)-induced IFN signaling pathway. MEFs were pre-treated with the indicated concentrations of HOIPIN-1, and then stimulated with 10 μg/ml poly(I:C) with HOIPIN-1 for the indicated period, and a qPCR analysis was performed. In (**b, d, e, f**), data are shown as means ± SEM, *n*=3, NS: not significant, **P* <0.05, ***P* <0.01, ****P* <0.001, *****P* <0.0001, one-way ANOVA with Tukey's post-hoc test.

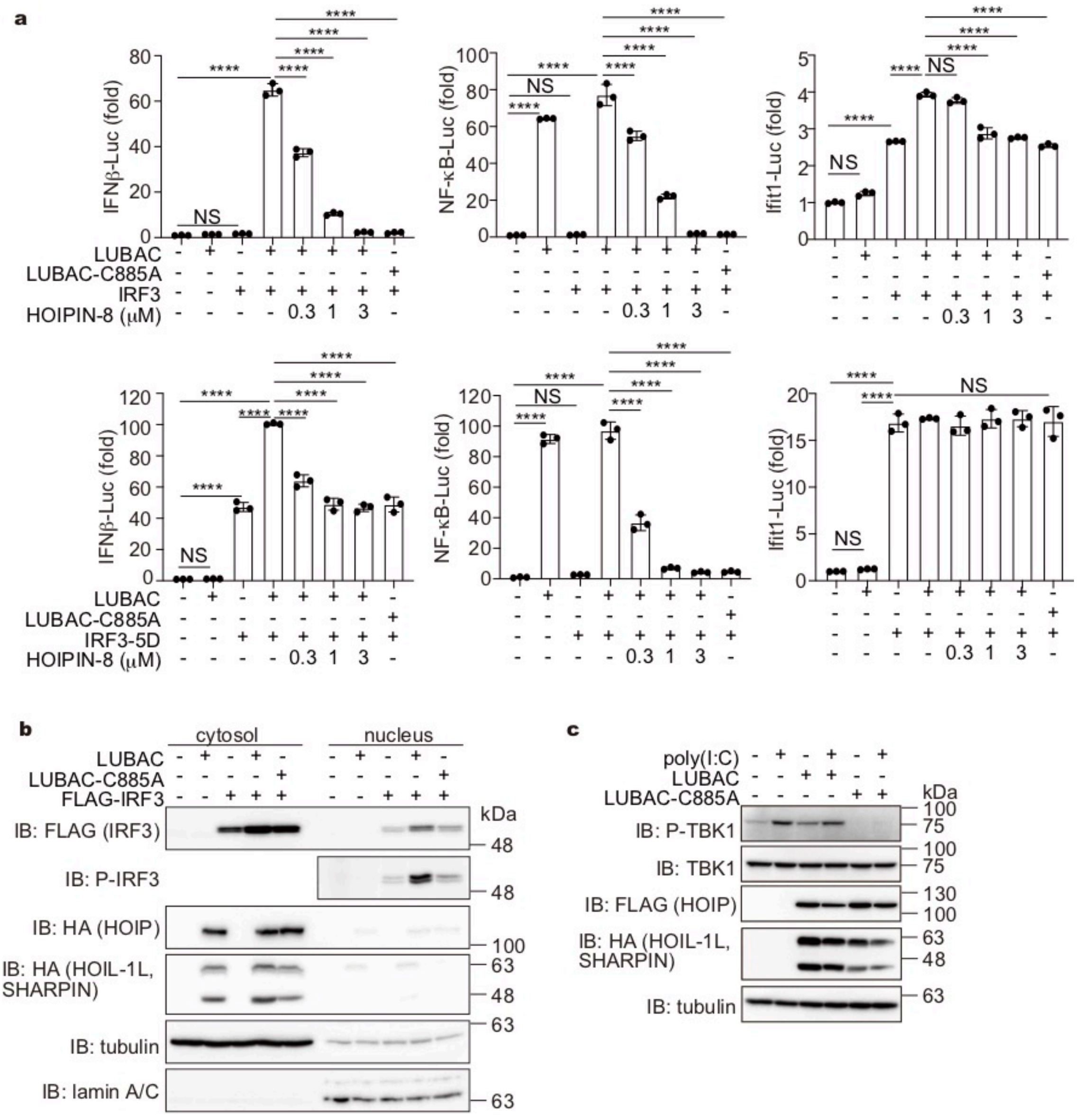

Supplementary Fig. 6 LUBAC activity is necessary for TBK1-mediated IRF3 activation in antiviral pathway. **a** HEK293T cells were transfected with wild-type HOIP with HOIL-1L, and SHARPIN (LUBAC), inactive C885A-mutant of HOIP with HOIL-1L, and SHARPIN (LUBAC-C885A), wild-type IRF3, or constitutively active mutant of IRF3 (IRF3-5D), and the luciferase reporter-containing promoter of IFNβ, NF-κB, or Ifit1, as indicated. Cells were treated with the indicated concentrations of HOIPIN-8, and the luciferase activity in the cell lysates was analyzed at 24 h post-transfection. Data are shown as means ± SEM, *n*=3, NS: not significant, *****P* <0.0001, one-way ANOVA with Tukey's post-hoc test. **b** LUBAC activates IRF3. HEK293T cells were transfected with the indicated

plasmids, and cell lysates were separated into cytosolic and nuclear fractions. The samples were then subjected to an immunoblotting analysis, using the indicated antibodies. **c** LUBAC enhances TBK1 activation. LUBAC or LUBAC-C885A-expressing cells were stimulated with 6 μg/ml poly(I:C), and cell lysates were immunoblotted with the indicated antibodies.

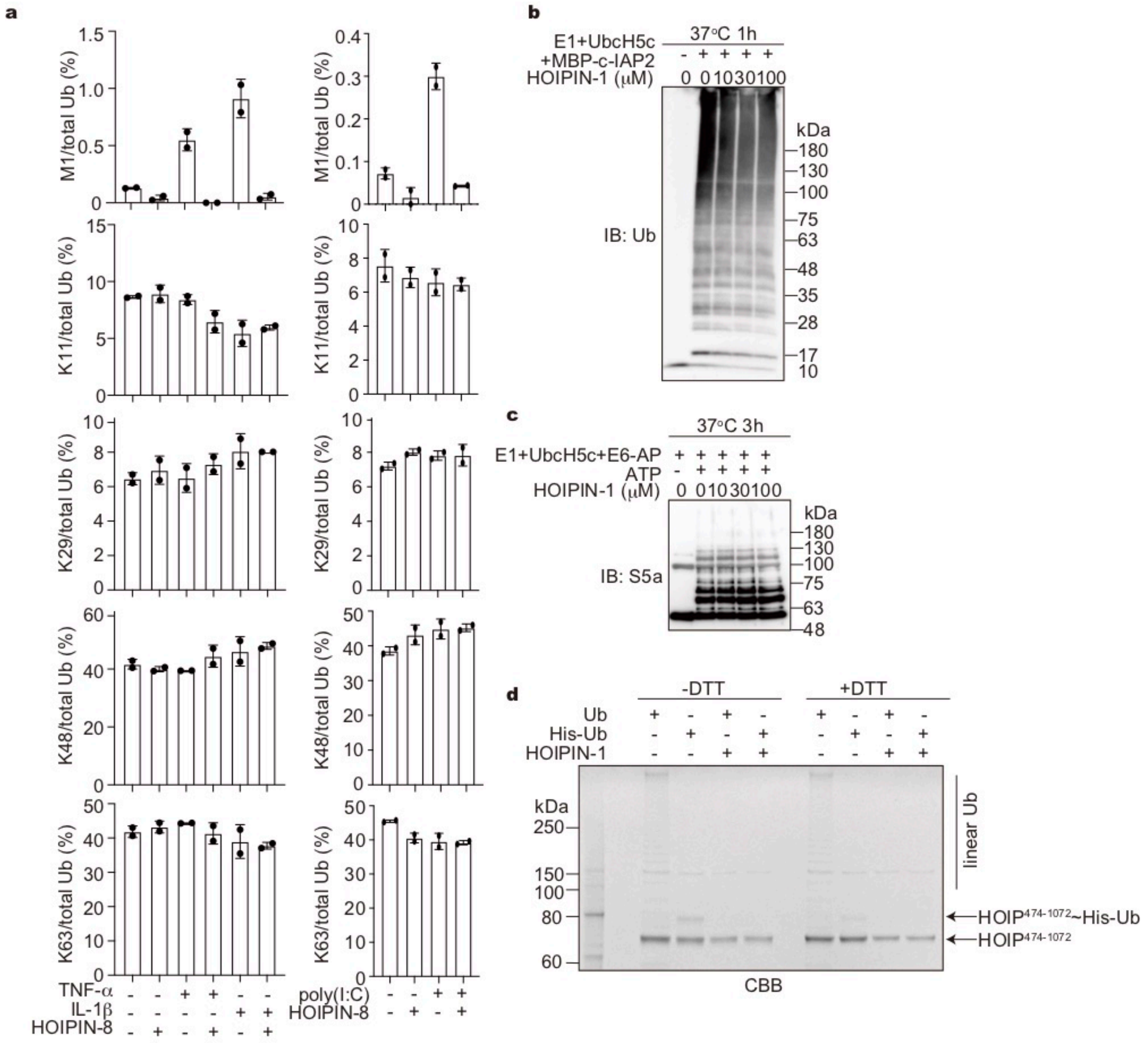

Supplementary Fig. 7 Selectivity of HOIPINs in LUBAC-mediated linear ubiquitination. **a** HOIPIN-8 selectively suppresses the intracellular linear ubiquitin level. A549 cells and BMDM were treated with TNF-α, IL-1β, or poly(I:C) as in Fig. 3a, and cell lysates were subjected to a quantitative analysis of the ubiquitin linkages by mass spectrometry. Relative amounts of ubiquitin linkages from two biological replicates are shown. **b, c** Effects of HOIPIN-1 on RING-type and HECT-type E3s. *In vitro* ubiquitination assays for MBP-cIAP2 (**b**) and E6-AP (**c**) were performed in the presence of various concentrations of HOIPIN-1, and immunoblotted with the indicated antibodies. **d** The thioester-bound ubiquitin on HOIP was suppressed by HOIPIN-1. *In vitro* ubiquitination was performed using 4.8 μg/ml E1, 9 μg/ml UbcH5C, and 16 μg/ml petit-LUBAC with 25 μg/ml WT-ubiquitin, 25 μg/ml His-ubiquitin, and/or 30 μM HOIPIN-1 as indicated. Samples

were electrophoresed in either the absence or presence of 50 mM DTT, and stained with Coomassie Brilliant Blue (CBB).

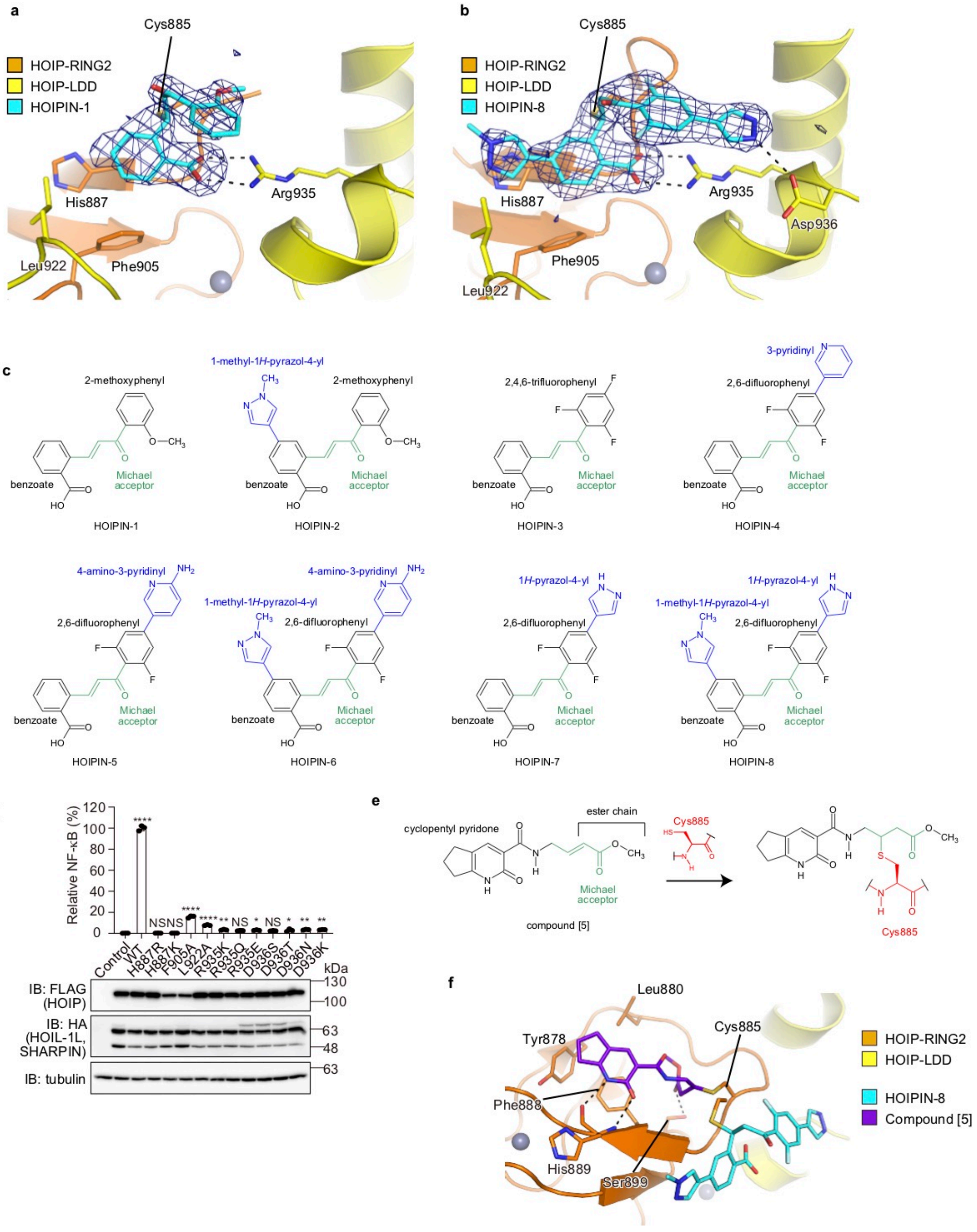

Supplementary Fig. 8 Structural basis for HOIPINs-mediated HOIP inactivation. **a, b** Structures of the HOIPIN-1- and HOIPIN-8-bound HOIP complexes. Crystal structures of the HOIP RING2-LDD domain complexed with HOIPIN-1 (**a**) or HOIPIN-8 (**b**) are indicated. Hydrogen bonds are indicated by dashed lines, and crucial residues for interactions with HOIPINs are shown. Simulated annealing

omit F_o – F_c maps, calculated with HOIPIN-1 or HOIPIN-8 removed, are shown as blue meshes contoured at 3 σ. **c** Chemical structures of the developed HOIPIN-1 derivatives[29]. Michael acceptors are colored green. Additional aromatic rings of the HOIPIN-1 derivatives are colored blue. **d** The relative NF-κB activity induced by HOIPINs-binding sites mutants of HOIP. The indicated HOIP mutants were expressed in HEK293T cells with HOIL-1L, SHARPIN, and an NF-κB luciferase reporter. Taking the NF-κB activity induced by wild-type (WT)-HOIP as 100%, the relative activities induced by the mutants are shown. Expression of LUBAC subunits was examined by immunoblotting, using the indicated antibodies. Data are shown as means ± SEM, n=3, NS: not significant, *P <0.05, **P <0.01, ****P <0.0001, one-way ANOVA with Tukey's post-hoc test. **e** Reaction mechanism of compound [5] with the active Cys885 in HOIP[27]. **f** Close-up view of the HOIPIN-8- or compound [5]-bound HOIP. Residues interacting with compound [5] are indicated.

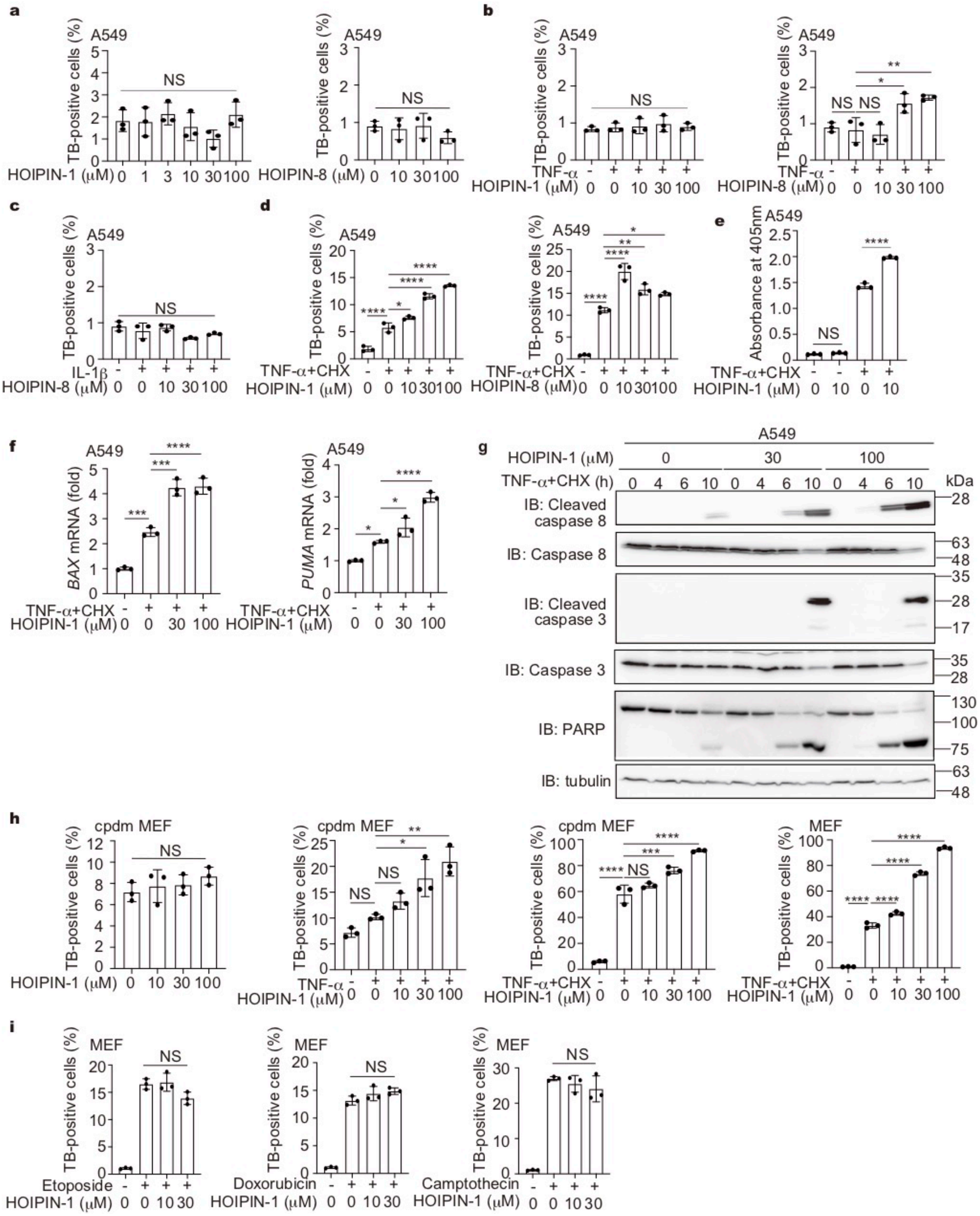

Supplementary Fig. 9 HOIPINs enhance the TNF-α-induced extrinsic apoptotic pathway. **a** HOIPINs show little cytotoxicity. A549 cells were treated with the indicated concentrations of HOIPIN-1 or HOIPIN-8 for 14 h, and a trypan blue dye exclusion assay was performed. **b** HOIPINs accelerate TNF-α-induced cell death. A549 cells were pre-treated with the indicated concentrations of HOIPIN-

1 or HOIPIN-8 for 30 min, and stimulated with 40 ng/ml TNF-α for 14 h. A trypan blue dye exclusion assay was then performed. **c** HOIPINs show no cytotoxicity in IL-1β-stimulated cells. A similar analysis as in (**b**) was performed, in the presence of 1 ng/ml IL-1β. **d** HOIPINs accelerate TNF-α+CHX-induced cell death. A549 cells were pre-treated with the indicated concentrations of HOIPIN-1 or HOIPIN-8 for 30 min, stimulated with 5 ng/ml TNF-α + 5 μg/ml CHX for 14 h, and analyzed as in (**b**). **e** HOIPIN-1 accelerates TNF-α-induced DNA fragmentation. A549 cells were treated as in (**d**), and cytoplasmic histone-associated DNA fragments were assessed by ELISA. **f** mRNA expression of apoptosis-associated factors. A549 cells were pre-treated with the indicated concentrations of HOIPIN-1 for 30 min, and then stimulated with 40 ng/ml TNF-α + 20 μg/ml CHX for 8 h. The mRNA levels of *BAX* and *PUMA* were then assessed by qPCR analyses. **g** Caspase activation in HOIPIN-1-treated cells. A549 cells were pre-treated with the indicated concentrations of HOIPIN-1, and then stimulated with 40 ng/ml TNF-α + 20 μg/ml CHX for the indicated period. The cell lysates were then immunoblotted with the depicted antibodies. **h** Effects of HOIPIN-1 on TNF-α-induced apoptosis in *Sharpin*-deficient *cpdm* MEF cells. WT- or *cpdm* MEF cells were treated with the indicated concentrations of HOIPIN-1, followed by a treatment with 10 ng/ml TNF-α + 5 μg/ml CHX for 8 h, and then a trypan blue dye exclusion assay was performed. **i** HOIPIN-1 has no effects on the intrinsic apoptotic pathway. MEFs were pre-treated with the indicated concentrations of HOIPIN-1, and then with genotoxic agents, such as 10 μM etoposide, 10 μM doxorubicin, or 2 μM camptothecin, for 14 h. A trypan blue dye exclusion assay was then performed. In (**a-f, h, i**), data are shown as means ± SEM, n=3, NS: not significant, *P<0.05, **P <0.01, ***P <0.001, ****P <0.0001, one-way ANOVA with Tukey's post-hoc test.

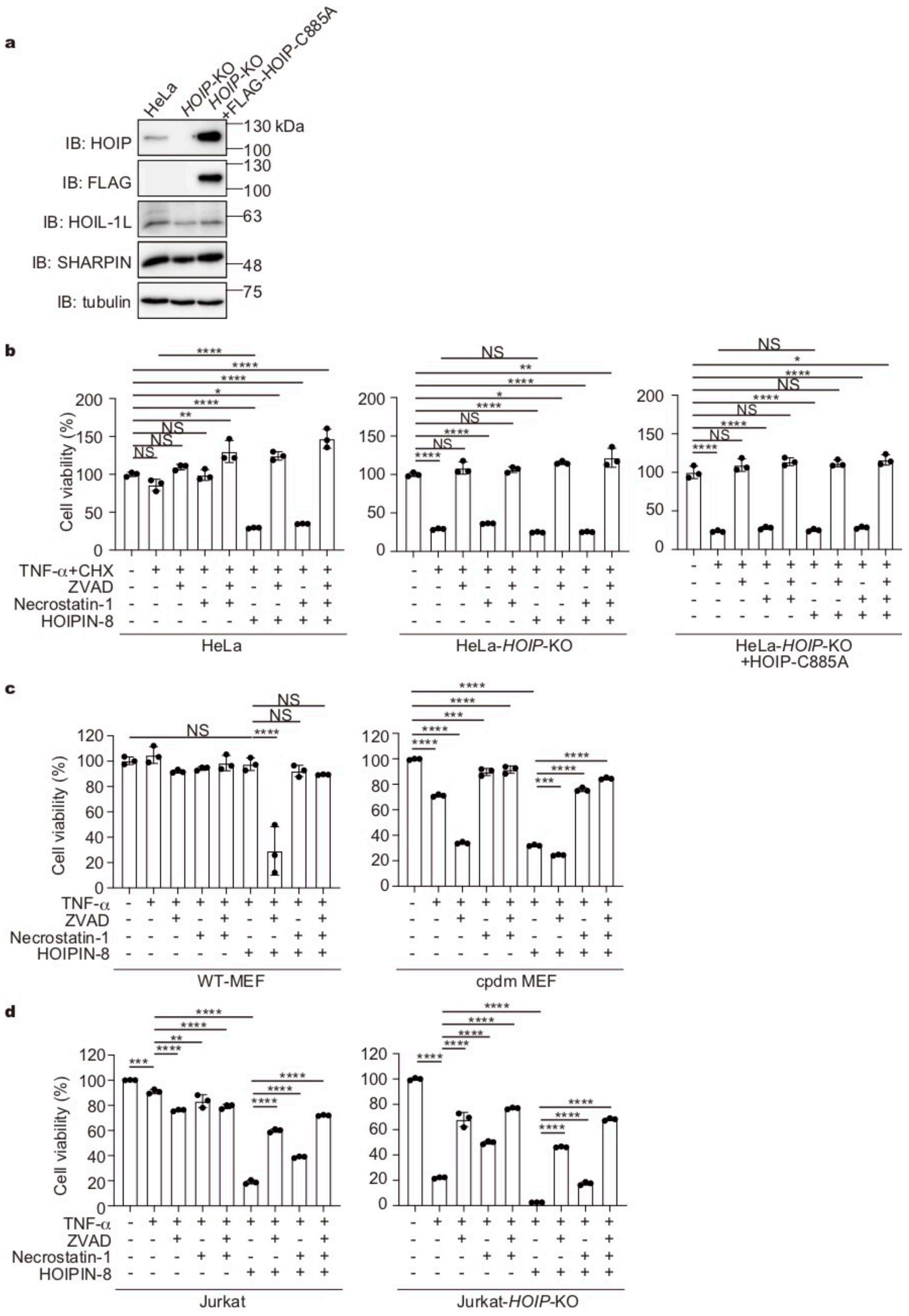

Supplementary Fig. 10 HOIPIN-8 accelerates TNF-α-induced cell death. **a** The expression of LUBAC subunits in parental HeLa cells, *HOIP*-deficient cells, and FLAG-HOIP-C885A mutant restored cells was examined by immunoblotting, using the indicated antibodies. **b** Effect of HOIPIN-8 on TNF-α+CHX-induced cell death in HeLa cells. Parental HeLa, *HOIP*-deficient, and C885A mutant restored

cells were treated with 5 ng/ml TNF-α + 5 μg/ml CHX, 20 μM ZVAD, 100 μM necrostain-1, and/or 3 μM HOIPIN-8 for 8 h, as indicated. Cell viability was assessed by a Cell-titer Glo assay, and taking the cell viability in the control as 100%, the relative cell viabilities are shown. **c** The effect of HOIPIN-8 on TNF-α alone-induced cell death in MEFs. Wild-type MEFs and *Sharpin*-deficient cpdm MEFs were treated with 10 ng/ml TNF-α, 20 μM ZVAD, 100 μM necrostain-1, and/or 10 μM HOIPIN-8 for 20 h, as indicated, and analyzed as in (**b**). **d** The effect of HOIPIN-8 on 100 ng/ml TNF-α alone-induced cell death in parental and *HOIP*-deficient Jurkat cells was analyzed as in (**c**). In (**b-d**), data are shown as means ± SEM, n=3, NS: not significant, *P<0.05, **P <0.01, ***P <0.001, ****P <0.0001, one-way ANOVA with Tukey's post-hoc test.

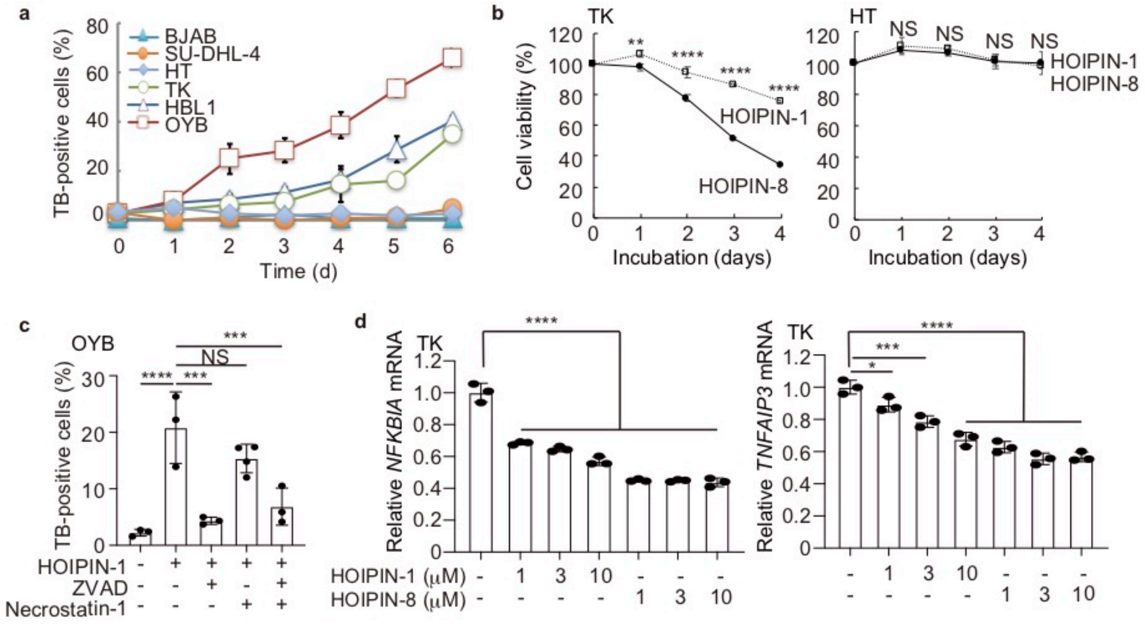

Supplementary Fig. 11 HOIPINs enhance cell death in ABC-DLBCL cell lines. **a** Enhanced cell death in ABC-DLBCL cells. ABC-DLBCL (TK, HBL1, and OYB) and GCB-DLBCL (BJAB, SU-DHL-4, and HT) cell lines were cultured in the presence of 10 μM HOIPIN-1, and dead cells were counted by a trypan blue dye exclusion assay. **b** HOIPIN-8 shows more potent inhibitory effects on ABC-DLBCL cells than HOIPIN-1. TK (ABC-DLBCL) and HT (GCB-DLBCL) cells were treated with DMSO, 3 μM HOIPIN-1 or 3 μM HOIPIN-8 for the indicated period. Taking the cell viabilities in the presence of DMSO as 100%, the relative cell viabilities of TK and HT cells were assessed by a CellTiter-Glo luminescent cell viability assay. **c** HOIPIN-1 accelerates apoptosis in ABC-DLBCL cells. OYB cells were pre-treated with 10 μM HOIPIN-1, and then treated with 20 μM ZVAD, and/or 100 μM necrostain-1 for 48 h, and a trypan blue dye exclusion assay was performed. **d** Suppression of NF-κB-target gene expression in ABC-DLBCL cells. TK cells were treated with the indicated concentrations of HOIPIN-1 or -8 for 24 h. The mRNA levels of *NFKBIA* (which encodes IκBα) and *TNFAIP3* (which encodes A20) were then assessed by qPCR analyses. In (**b-d**), data are shown as means ± SEM, *n*=3, NS: not significant, **P* <0.05, ***P* <0.01, ****P* <0.001, *****P* <0.0001, one-way ANOVA with Tukey's post-hoc test.

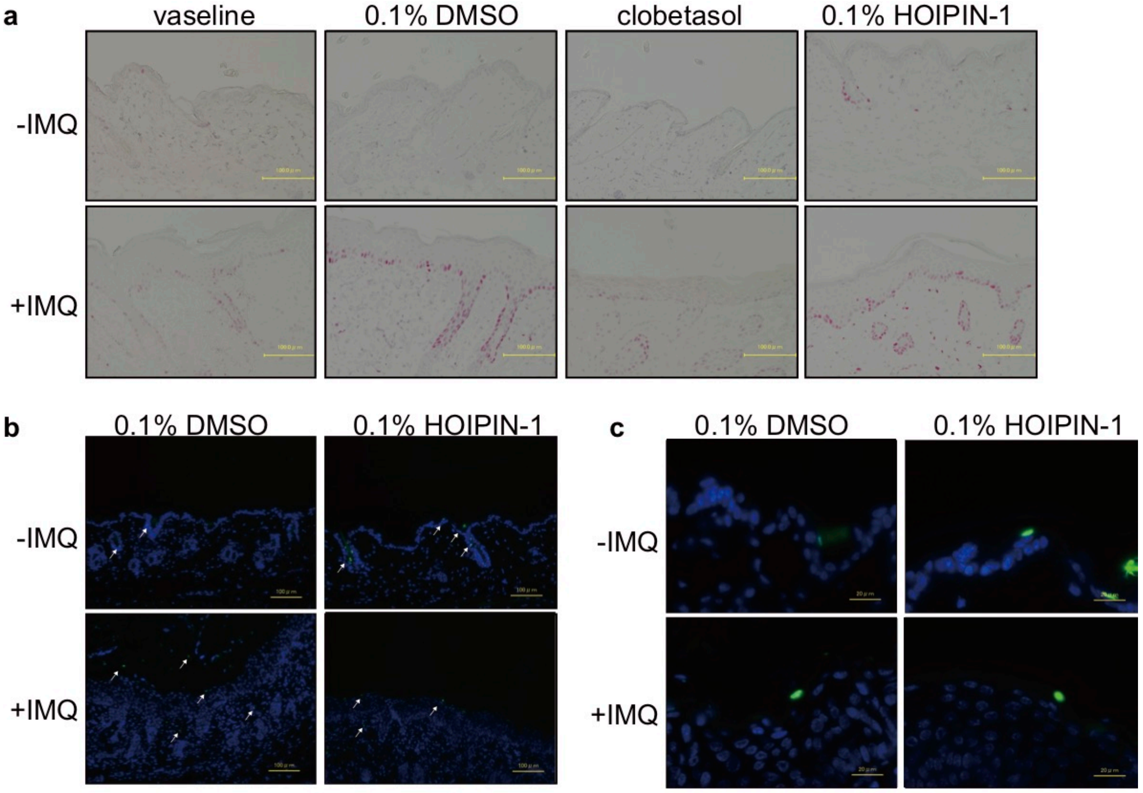

d

	TUNEL-positive cells/total counted cells			
IMQ	-	+	-	+
HOIPIN-1	-	-	+	+
mouse #1	53/7,347	23/10,981	17/3,144	68/15,987
mouse #2	82/5,558	22/12,145	12/3,767	23/12,409
mouse #3	42/7,449	20/11,160	10/2,899	34/13,446

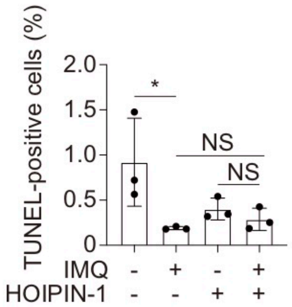

Supplementary Fig. 12 Effects of HOIPIN-1 on epidermal growth and cell death. **a** Ki-67 expression in imiquimod (IMQ)-model mice. Sections from mouse back skin samples, treated as in Figure 7c, were stained with an anti-Ki67 antibody, a biotinylated secondary antibody, alkaline phosphatase-conjugated avidin, and FAST Red (Sigma). Bars, 100 μm. **b, c** HOIPIN-1 shows minimal effects on psoriasis-associated apoptosis. Low (**b**) and high (**c**) magnification images of sections from mouse back skin samples, treated as indicated, were stained with TUNEL (green), and the nuclei were stained with DAPI (blue). *Arrows*, TUNEL-positive cells. Bars, 100 μm (**b**) and 20 μm (**c**). **d** IMQ and HOIPIN-1 exerted minimal effects on cell death in the psoriasis model mice. TUNEL-positive cells in the sections treated as indicated were counted. Data are shown as means ±

SEM, *n*=3, NS: not significant, *P <0.05, one-way ANOVA with Tukey's post-hoc test.

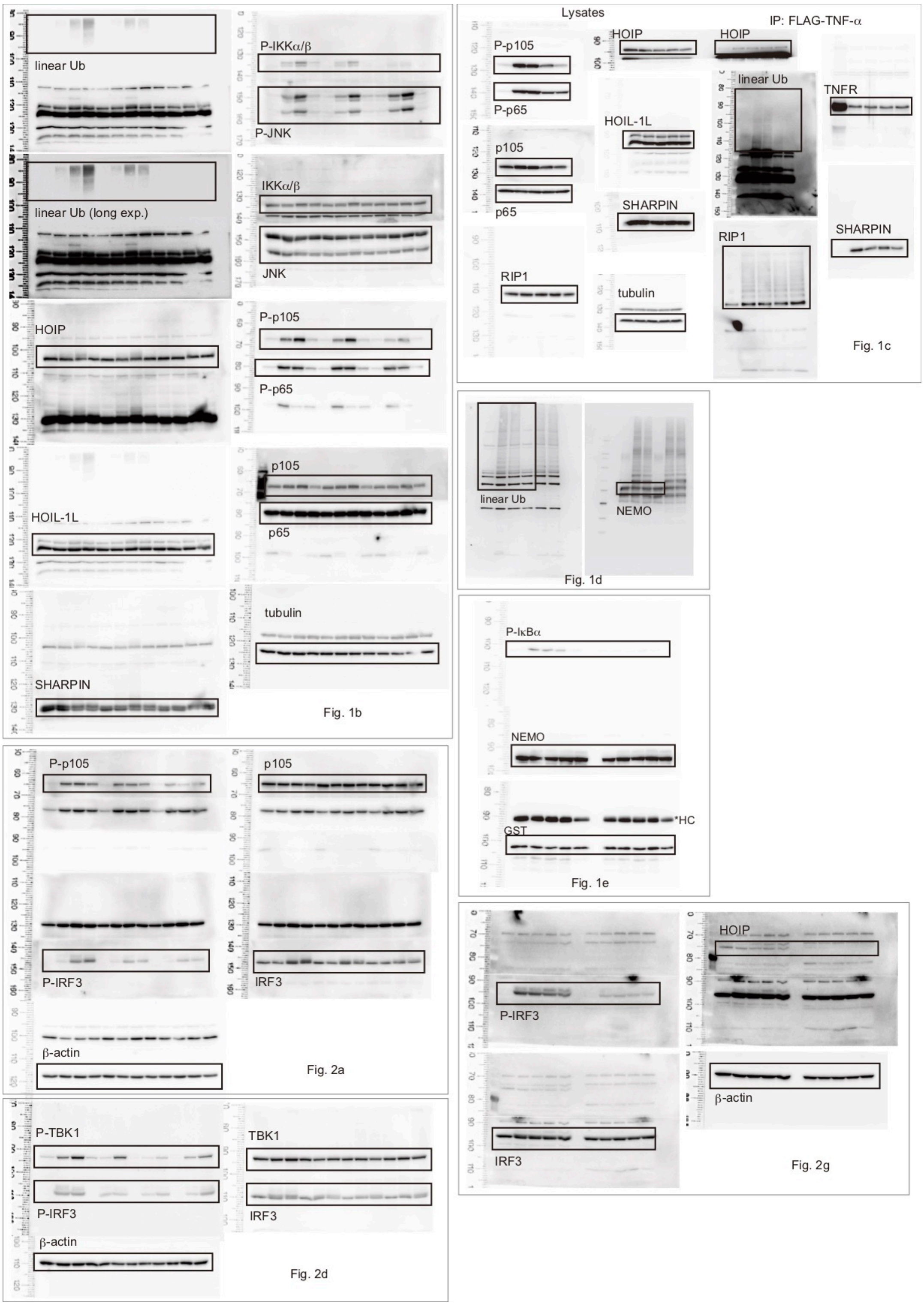

Supplementary Fig. 13 Uncropped blots for Fig. 1 and 2.

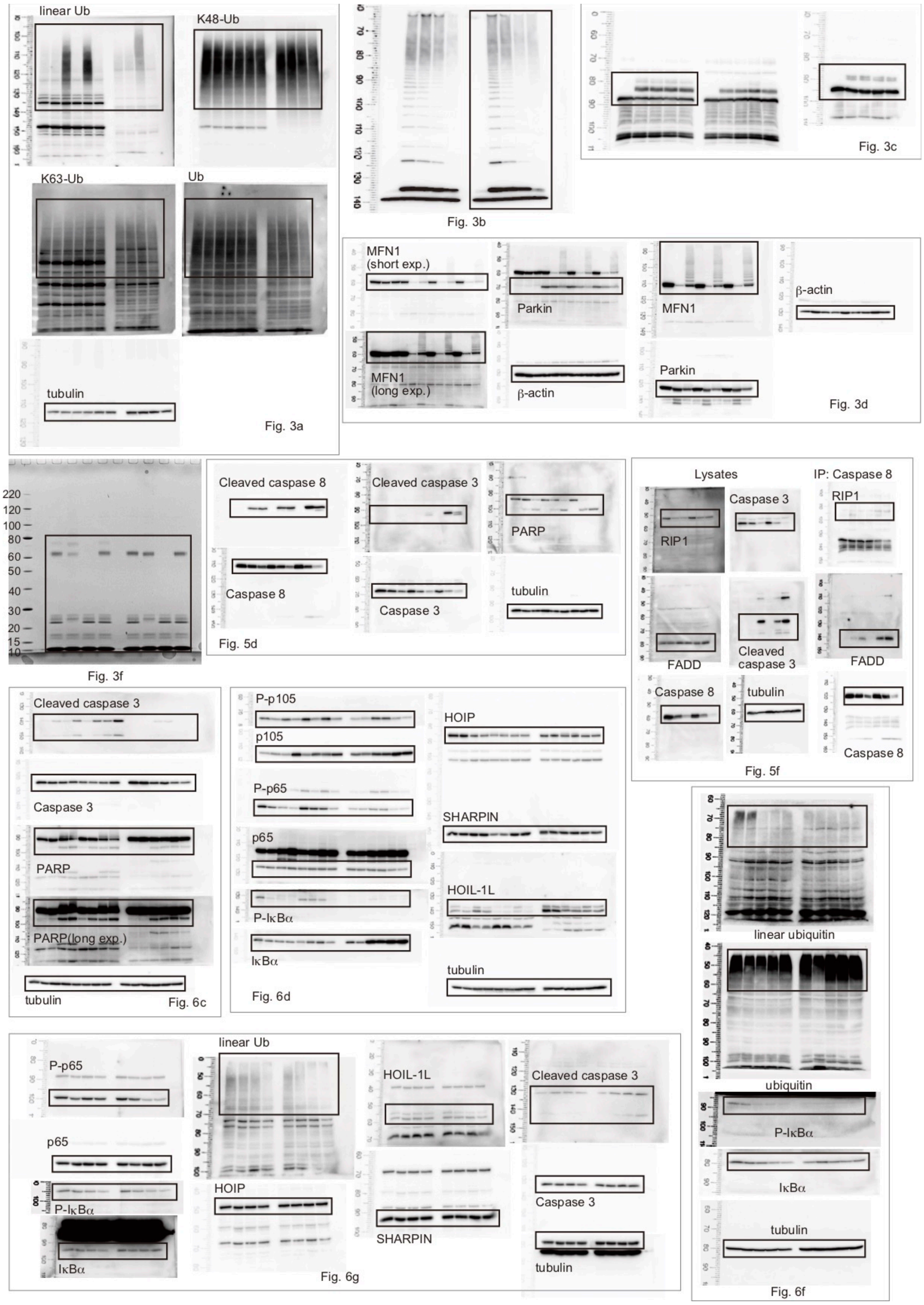

Supplementary Fig. 14 Uncropped blots for Fig. 3, 5, and 6.

Supplementary Table 1 Inhibitory effect of HOIPINs on the growth of various cells. Cells were treated with various concentrations of HOIPIN-1 or -8 for 72 h, and the viabilities were analyzed by a CellTiter-Glo luminescent cell viability assay. IC_{50} values of HOIPINs are represented.

Cells	HOIPIN-1 (IC_{50}, μM)	HOIPIN-8 (IC_{50}, μM)	Cell type
A549	>100	>100	lung carcinoma
HEK293T	40.3	70.3	embryonic kidney
WT-MEF	>100	49.7	fibroblast
cpdm MEF	>100	51.8	fibroblast
Jurkat	20.6	>100	T lymphocyte
HeLa	33.7	>100	cervical adenocarcinoma
TK	9.7	3.7	ABC-DLBCL
DLBCL2	11.1	4.7	ABC-DLBCL
OYB	16.0	14.4	ABC-DLBCL
HBL1	16.4	16.7	ABC-DLBCL
BJAB	52.2	>30	GCB-DLBCL
SU-DHL-4	46.9	>30	GCB-DLBCL
HT	48.2	>30	GCB-DLBCL
KM-H2	9.6	40.7	Hodgkin's lymphoma
Karpas1160P	11.0	27.6	non-Hodgkin's lymphoma

Supplementary Table 2 Primers used in this study.

REAGENT or RESOURCE	SOURCE	IDENTIFIER
Oligonucleotides		
For human		
IL-6 sense: 5'-AGCCACTCACCTCTTCAGAAC-3'	Nakazawa, S. et al., 2016	N/A
IL-6 anti-sense: 5'-GCCTCTTTGCTGCTTTCACAC-3'	Nakazawa, S. et al., 2016	N/A
ICAM1 sense: 5'-GTGGTAGCAGCCGCAGT-3'	Nakazawa, S. et al., 2016	N/A
ICAM1 anti-sense: 5'-TTCGGTTTCATGGGGGT-3'	Nakazawa, S. et al., 2016	N/A
NFKBIA sense: 5'-CGGGCTGAAGAAGGAGCGGC-3'	Nakazawa, S. et al., 2016	N/A
NFKBIA anti-sense: 5'-ACGAGTCCCCGTCCTCGGTG-3'	Nakazawa, S. et al., 2016	N/A
BCL-2a sense: 5'-CAGGAGAATGGATAAGGCAAA-3'	Nakazawa, S. et al., 2016	N/A
BCL-2a anti-sense: 5'-CCAGCCAGATTTAGGTTCAAA-3'	Nakazawa, S. et al., 2016	N/A
TNF-α sense: 5'-GCCGCATCGCCGTCTCCTAC-3'	This study	N/A
TNF-α anti-sense: 5'-CCTCAGCCCCCTCTGGGGTC-3'	This study	N/A
TNFAIP3 sense: 5'-CATGCATGCCACTTCTCAGT-3'	Nakazawa, S. et al., 2016	N/A
TNFAIP3 anti-sense: 5'-CATGGGTGTGTCTGTGGAG-3'	Nakazawa, S. et al., 2016	N/A
BAX sense: 5'-GTCGTCTTCTCTATTTTGC-3'	This study	N/A
BAX anti-sense: 5'-GCAGCAAGTCTAATGTGCAG -3'	This study	N/A
PUMA sense: 5'-TTGTCTGCACGATGTTGCATTCTGGA-3'	This study	N/A
PUMA sense: 5'-GCCAGGGCCAGTCTCTAGGA-3'	This study	N/A
GAPDH sense: 5'-AGCAACAGGGTGGTGGAC-3'	Nakazawa, S. et al., 2016	N/A
GAPDH anti-sense: 5'-GTGTGGTGGGGGACTGAG-3'	Nakazawa, S. et al., 2016	N/A
For mouse		
Bcl2l1 sense: 5'-GGTGAGTCGGATTGCAAGTT-3'	This study	N/A
Bcl2l1 anti-sense: 5'-GCTGCATTGTTCCCGTAGAG-3'	This study	N/A
Tnf-α sense: 5'-TAGCCAGGAGGGAGAACAGA-3'	This study	N/A
Tnf-α anti-sense: 5'-TTTTCTGGAGGGAGATGTGG-3'	This study	N/A
Il-6 sense: 5'-CTGATGCTGGTGACAACCAC-3'	This study	N/A
Il-6 anti-sense: 5'-TCCACGATTTCCCAGAGAAC-3'	This study	N/A
Il-1β sense: 5'-CCCTGCAGCTGGAGAGTGTGGA-3'	This study	N/A
Il-1β anti-sense: 5'-TGTGCTCTGCTTGTGAGGTGCTG-3'	This study	N/A
Cxcl10 sense: 5'-CCAAGTGCTGCCGTCATTTTC-3'	This study	N/A
Cxcl10 anti-sense: 5'-GGCTCGCAGGGATGATTCAA-3'	This study	N/A
Tnfaip3 sense: 5'-GCTTTCGCAGAGGCAGTAACAG-3'	This study	N/A
Tnfaip3 anti-sense: 5'-AGCAAGTGCAGGAAAGCTGGCT-3'	This study	N/A
Icam1 sense: 5'-CCCACGCTACCTCTGCTC-3'	This study	N/A
Icam1 anti-sense: 5'-GATGGATACCTGAGCATCACC-3'	This study	N/A
Ifnb1 sense: 5'-CCCTATGGAGATGACGGAGA-3'	This study	N/A
Ifnb1 anti-sense: 5'-CTGTCTGCTGGTGGAGTTCA-3'	This study	N/A
Ifit1 sense: 5'-ACCATGGGAGAGAATGCTGAT-3'	This study	N/A
Ifit1 anti-sense: 5'-GCCAGGAGGTTGTGC-3'	This study	N/A
Isg15 sense: 5'- GGAACGAAAGCGGCCACAGCA-3'	This study	N/A
Isg15 anti-sense: 5'- CCTCCATGGGCCTTCCCTCGA-3'	This study	N/A
Gapdh sense: 5'-TTTGGCATTGTGGAAGGGCTCAT-3'	This study	N/A
Gapdh anti-sense: 5'-CACCAGTGGATGCAGGGATGATGT-3'	This study	N/A
TaqMan Gene Expression Assays		
Il17a	Applied Biosystems	Cat# Mm00439618_m1
Il17f	Applied Biosystems	Cat# Mm00521423_m1
Il22	Applied Biosystems	Cat# Mm01226722_g1
Il23a	Applied Biosystems	Cat# Mm00518984_m1
Gapdh	Applied Biosystems	Cat# Mm99999915_g1
