# Supplementary material for: Molecular bases for HOIPINs-mediated inhibition of LUBAC and innate immune responses
Source: Commun Biol. 2020 Apr 3;3:163. doi: 10.1038/s42003-020-0882-8 (PMC7125101; doi:10.1038/s42003-020-0882-8)
Supplement: Supplementary file 2 — Description of Additional Supplementary Files [file 42003_2020_882_MOESM2_ESM.pdf]

Description of additional supplementary items

Supplementary Data 1 (excel file) contains the source data underlying the graphs and charts presented in the article, including Figures 1f, 2b, 2c, 2e, 2f, 2h, 2i, 4f, 5a, 5b, 5c, 5e, 6a, 6b, 6e, 7b, 7d, and 7e.
