# Supplementary material for: Molecular bases for HOIPINs-mediated inhibition of LUBAC and innate immune responses
Source: Commun Biol. 2020 Apr 3;3:163. doi: 10.1038/s42003-020-0882-8 (PMC7125101; doi:10.1038/s42003-020-0882-8)
Supplement: Supplementary file 4 — Reporting Summary [file 42003_2020_882_MOESM4_ESM.pdf]

nature research

Corresponding author(s): Fuminori Tokunaga

Last updated by author(s): Feb. 17, 2020

Reporting Summary

Nature Research wishes to improve the reproducibility of the work that we publish. This form provides structure for consistency and transparency in reporting. For further information on Nature Research policies, see Authors & Referees and the Editorial Policy Checklist.

Statistics

For all statistical analyses, confirm that the following items are present in the figure legend, table legend, main text, or Methods section.

n/a	Confirmed	
☐	☒	The exact sample size (*n*) for each experimental group/condition, given as a discrete number and unit of measurement
☐	☒	A statement on whether measurements were taken from distinct samples or whether the same sample was measured repeatedly
☐	☒	The statistical test(s) used AND whether they are one- or two-sided<br>*Only common tests should be described solely by name; describe more complex techniques in the Methods section.*
☒	☐	A description of all covariates tested
☒	☐	A description of any assumptions or corrections, such as tests of normality and adjustment for multiple comparisons
☐	☒	A full description of the statistical parameters including central tendency (e.g. means) or other basic estimates (e.g. regression coefficient) AND variation (e.g. standard deviation) or associated estimates of uncertainty (e.g. confidence intervals)
☐	☒	For null hypothesis testing, the test statistic (e.g. F, t, r) with confidence intervals, effect sizes, degrees of freedom and P value noted<br>*Give P values as exact values whenever suitable.*
☒	☐	For Bayesian analysis, information on the choice of priors and Markov chain Monte Carlo settings
☒	☐	For hierarchical and complex designs, identification of the appropriate level for tests and full reporting of outcomes
☒	☐	Estimates of effect sizes (e.g. Cohen's *d*, Pearson's *r*), indicating how they were calculated

Our web collection on statistics for biologists contains articles on many of the points above.

Software and code

Policy information about availability of computer code

Data collection	ImageQuant TL, Fusion Capt17, StepOne Software v. 2.1
Data analysis	Graph Pad Prism 8.3.0, MS Excel 16.31, Adobe Illustrator 12.0.4, Pymol, HKL2000, CCP4, Phenix

For manuscripts utilizing custom algorithms or software that are central to the research but not yet described in published literature, software must be made available to editors/reviewers. We strongly encourage code deposition in a community repository (e.g. GitHub). See the Nature Research guidelines for submitting code & software for further information.

Data

Policy information about availability of data

All manuscripts must include a data availability statement. This statement should provide the following information, where applicable:

- Accession codes, unique identifiers, or web links for publicly available datasets
- A list of figures that have associated raw data
- A description of any restrictions on data availability

X-ray crystal structure coordinates are available from Protein Data Bank under accession code 6KC5 and 6KC6. RNA sequencing data has been deposited in SRA database under BioProject ID PRJDB9322.

Field-specific reporting

Please select the one below that is the best fit for your research. If you are not sure, read the appropriate sections before making your selection.

☒ Life sciences ☐ Behavioural & social sciences ☐ Ecological, evolutionary & environmental sciences

Life sciences study design

All studies must disclose on these points even when the disclosure is negative.

Sample size	No sample size calculation was performed, but sample size was chosen based on the consistency and magnitude of measured differences between groups.
Data exclusions	No data were excluded from the analyses.
Replication	All attempts replicated similar results. We have repeated each experiment in the manuscript at least twice to ensure consistent results. We have also provided protocol on methods section.
Randomization	No randomization was used in this study.
Blinding	No blinding was done in this study.

Reporting for specific materials, systems and methods

We require information from authors about some types of materials, experimental systems and methods used in many studies. Here, indicate whether each material, system or method listed is relevant to your study. If you are not sure if a list item applies to your research, read the appropriate section before selecting a response.

Materials & experimental systems

n/a	Involved in the study	
☐	☒	Antibodies
☐	☒	Eukaryotic cell lines
☒	☐	Palaeontology
☐	☒	Animals and other organisms
☒	☐	Human research participants
☒	☐	Clinical data

Methods

n/a	Involved in the study	
☒	☐	ChIP-seq
☒	☐	Flow cytometry
☒	☐	MRI-based neuroimaging

Antibodies

Antibodies used

The following antibodies were used for Western blot and cell stimulation: parkin (Cell Signaling, 2132), P-IκBα (Cell Signaling, 9246), IκBα (Cell Signaling, 4812), P-p105 (Cell Signaling, 4806), p105 (Cell Signaling, 3035), P-p65 (Cell Signaling, 3033), p65 (Cell Signaling, 8242), P-IKKα/β (Cell Signaling, 2697), P-JNK (Cell Signaling, 4668), JNK (Cell Signaling, 9252), p100/p52 (Cell Signaling, 4882), P-TBK1 (Cell Signaling, 5483), TBK1 (Cell Signaling, 3504), P-IRF3 (Cell Signaling, 4947 and 37829), IRF3 (Cell Signaling, 4302), caspase 8 (Cell Signaling, 4790), cleaved caspase 8 (Cell Signaling, 9496), caspase 3 (Cell Signaling, 9662), cleaved caspase 3 (Cell Signaling, 9661), PARP (Cell Signaling, 9542), GST (Cell Signaling, 2622), MBP (Cell Signaling, 2396), Lamin A/C (Cell Signaling, 4777), MFN1 (Cell Signaling, 14739), P-TAK1 (Cell Signaling, 4508), TAK1 (Cell Signaling, 5206), HOIL-1L (Santa Cruz Biotech, sc-49718), OPTN (Santa Cruz Biotech, sc-166576), ubiquitin (P4D1) (Santa Cruz Biotech, sc-8017), TNFR1 (Santa Cruz Biotech, sc-7895), IKKα/β (Santa Cruz Biotech, sc-7607), NEMO (Santa Cruz Biotech, sc-8330), β-actin (Santa Cruz Biotech, sc-47778), caspase 8 (Santa Cruz Biotech, sc-6136), RIP1 (BD Biosciences, 610458), FADD (BD Biosciences, 610399), CD3 (BD Biosciences, 555337), CD28 (BD Biosciences, 555726), linear ubiquitin (LUB9) (Millipore, MABS451), tubulin (Cedarlane, CLT9002), NEMO (MBL, K0159-3), HOIP (Abcam, ab125189), SHARPIN (Proteintech, 14626-1-AP), HA (Roche, 11867423001), DYKDDDDK (1E6; HRP-Conjugate) (Wako, 015-22391), Ki67 (Dako, M7240), CD40 (HM40-3) (eBioscience, 16-0402-85), LTβR(3C8) (eBioscience, 16-5671-82), IgM (Southern Biotechnology, 2022-01), LTβR(4H8 WH2) (AdipoGen, AG-20B-0008).

Validation

All primary antibodies were used in this study are commercial, and validated from the manufacture's website as follows: parkin (https://en.cellsignal.jp/products/primary-antibodies/parkin-antibody/2132), P-IκBα (https://en.cellsignal.jp/products/primary-antibodies/phospho-ikba-ser32-36-5a5-mouse-mab/9246), IκBα (https://en.cellsignal.jp/products/primary-antibodies/ikba-44d4-rabbit-mab/4812), P-p105 (https://en.cellsignal.jp/products/primary-antibodies/phospho-nf-kb-p105-ser933-18e6-rabbit-mab/4806), p105 (https://en.cellsignal.jp/products/primary-antibodies/nf-kb1-p105-p50-antibody/3035), P-p65 (https://en.cellsignal.jp/products/primary-antibodies/phospho-nf-kb-p65-ser536-93h1-rabbit-mab/3033), p65 (https://en.cellsignal.jp/products/primary-antibodies/nf-kb-p65-d14e12-xp-rabbit-mab/8242), P-IKKα/β (https://en.cellsignal.jp/products/primary-antibodies/phospho-ikka-b-ser176-180-16a6-rabbit-mab/2697), P-JNK (https://en.cellsignal.jp/products/primary-antibodies/phospho-sapk-jnk-thr183-tyr185-81e11-rabbit-mab/4668), JNK (https://en.cellsignal.jp/products/primary-antibodies/sapk-jnk-antibody/9252), p100/p52 (https://en.cellsignal.jp/products/primary-antibodies/nf-kb2-p100-p52-antibody/4882), P-TBK1 (https://en.cellsignal.jp/products/primary-antibodies/phospho-tbk1-nak-ser172-d52c2-xp-rabbit-mab/5483), TBK1 (https://en.cellsignal.jp/products/primary-antibodies/tbk1-nak-d1b4-rabbit-mab/3504), P-IRF3 (https://en.cellsignal.jp/products/primary-antibodies/phospho-irf-3-ser396-4d4g-rabbit-mab/4947 and https://en.cellsignal.jp/products/primary-antibodies/phospho-irf-3-ser386-e7j8g-xp-rabbit-mab/37829), IRF3 (https://en.cellsignal.jp/products/primary-antibodies/irf-3-d83b9-rabbit-mab/4302), caspase 8 (https://en.cellsignal.jp/products/primary-antibodies/caspase-8-d35g2-rabbit-mab/4790), cleaved caspase 8 (https://en.cellsignal.jp/products/primary-antibodies/cleaved-caspase-8-asp391-18c8-rabbit-mab/9496), caspase 3

(https://en.cellsignal.jp/products/primary-antibodies/caspase-3-antibody/9662), cleaved caspase 3 (https://en.cellsignal.jp/products/primary-antibodies/cleaved-caspase-3-asp175-antibody/9661), PARP (https://en.cellsignal.jp/products/primary-antibodies/parp-antibody/9542), GST (https://en.cellsignal.jp/products/primary-antibodies/gst-antibody/2622), MBP (https://en.cellsignal.jp/products/primary-antibodies/mbp-tag-8g1-mouse-mab/2396), Lamin A/C (https://en.cellsignal.jp/products/primary-antibodies/lamin-a-c-4c11-mouse-mab/4777), MFN1 (https://en.cellsignal.jp/products/primary-antibodies/mitofusin-1-d6e2s-rabbit-mab/14739), P-TAK1 (https://en.cellsignal.jp/products/primary-antibodies/phospho-tak1-thr184-187-90c7-rabbit-mab/4508), TAK1 (https://en.cellsignal.jp/products/primary-antibodies/tak1-d94d7-rabbit-mab/5206), HOIL-1L (https://antibodyregistry.org/search.php?q=AB_2175281), OPTN (https://www.scbt.com/scbt/product/optineurin-antibody-c-2?productCanUrl=optineurin-antibody-c-2&_requestid=96946), ubiquitin (P4D1) (https://www.scbt.com/scbt/product/ub-antibody-p4d1?requestFrom=search), TNFR1 (https://www.scbt.com/scbt/product/tnf-r1-antibody-h-271?requestFrom=search), IKKα/β (https://www.scbt.com/scbt/product/ikkalpha-beta-antibody-h-470?requestFrom=search), NEMO (https://www.scbt.com/scbt/product/ikkgamma-antibody-fl-419?requestFrom=search), β-actin (https://www.scbt.com/scbt/product/beta-actin-antibody-c4?requestFrom=search), RIP1 (http://www.bdbiosciences.com/us/applications/research/apoptosis/purified-antibodies/purified-mouse-anti-rip-38rip/p/610458), FADD (http://www.bdbiosciences.com/us/applications/research/apoptosis/purified-antibodies/purified-mouse-anti-human-fadd-1fadd/p/610399), CD3 (http://www.bdbiosciences.com/us/applications/research/t-cell-immunology/th-1-cells/surface-markers/human/purified-mouse-anti-human-cd3-hit3a/p/555337), CD28 (http://www.bdbiosciences.com/us/applications/research/t-cell-immunology/regulatory-t-cells/surface-markers/human/purified-mouse-anti-human-cd28-cd282/p/555726), linear ubiquitin (LUB9) (http://www.merckmillipore.com/JP/en/product/Anti-Linear-Ubiquitin-Antibody-clone-LUB9,MM_NF-MABS451), tubulin (https://www.cedarlanelabs.com/products/detail?code=CLT9002), NEMO (https://www.mblintl.com/assets/K0157-3.pdf), HOIP (https://www.abcam.co.jp/rnf31hoip-antibody-ab125189.html), SHARPIN (https://www.ptglab.com/Products/SHARPIN-Antibody-14626-1-AP.htm), HA (https://www.sigmaaldrich.com/content/dam/sigma-aldrich/docs/Roche/Bulletin/1/roahahabul.pdf), DYKDDDDK (https://labchem-wako.fujifilm.com/us/product/detail/W01W0101-2239.html), Ki67 (https://www.citeab.com/antibodies/2390690-m7240-ki-67-antigen-concentrate), CD40 (HM40-3) (https://www.thermofisher.com/antibody/product/CD40-Antibody-clone-HM40-3-Monoclonal/16-0402-82), LTβR(3C8) (https://www.thermofisher.com/antibody/product/Lymphotoxin-beta-Receptor-Antibody-clone-eBio3C8-3C8-Monoclonal/16-5671-82), IgM (https://www.southernbiotech.com/?catno=2022-01&type=Polyclonal#&panel1-1&panel2-1), LTβR(4H8 WH2) (https://adipogen.com/ag-20b-0008-anti-lt-beta-r-mouse-mab-4h8-wh2.html/).

Eukaryotic cell lines

Policy information about cell lines

Cell line source(s)	A549, HEK293T, HeLa, BJAB, and Jurkat cells were obtained from ATCC; TK, DLBCL2, OYB, HBL1, SU-DHL-4, and HT were provided by Hitoshi Ohno (Tenri Hospital and Tenri Institute of Medical Research).
Authentication	Common cell lines were identified by their morphology and STR DNA profiling. Specific cell lines used in this study were authenticated by PCR or mutational status.
Mycoplasma contamination	All cell lines were tested and negative for mycoplasma contamination.
Commonly misidentified lines (See ICLAC register)	The cell lines used in this study are not in the commonly misidentified lines list.

Animals and other organisms

Policy information about studies involving animals; ARRIVE guidelines recommended for reporting animal research

Laboratory animals	8-week old female BALB/c mice were used for experiments.
Wild animals	The study did not involve wild animals.
Field-collected samples	The study did not involve samples collected from the field.
Ethics oversight	All animal experiments were performed according to the protocol that approved by Osaka City University.

Note that full information on the approval of the study protocol must also be provided in the manuscript.
